# Stabilization of Resveratrol in Blood Circulation by Conjugation to mPEG and mPEG-PLA Polymers: Investigation of Conjugate Linker and Polymer Composition on Stability, Metabolism, Antioxidant Activity and Pharmacokinetic Profile

**DOI:** 10.1371/journal.pone.0118824

**Published:** 2015-03-23

**Authors:** Basavaraj Siddalingappa, Heather A. E. Benson, David H. Brown, Kevin T. Batty, Yan Chen

**Affiliations:** 1 School of Pharmacy, CHIRI Biosciences, Curtin University, Perth, Western Australia; 2 Department of Chemistry, Curtin University, Perth, Western Australia; UMR INSERM U866, FRANCE

## Abstract

Resveratrol is naturally occurring phytochemical with diverse biological activities such as chemoprevention, anti-inflammatory, anti-cancer, anti-oxidant. But undergoes rapid metabolism in the body (half life 0.13h). Hence Polymer conjugation utilizing different chemical linkers and polymer compositions was investigated for enhanced pharmacokinetic profile of resveratrol. Ester conjugates such as α-methoxy-ω-carboxylic acid poly(ethylene glycol) succinylamide resveratrol (MeO-PEG**N**-Succ-RSV) (2 and 20 kDa); MeO-PEG succinyl ester resveratrol (MeO-PEG**O**-Succ-RSV) (2 kDa); α-methoxy poly(ethylene glycol)-co-polylactide succinyl ester resveratrol (MeO-PEG-PLAO-Succ-RSV) (2 and 6.6kDa) were prepared by carbodiimide coupling reactions. Resveratrol-PEG ethers (2 and 5 kDa) were synthesized by alkali-mediated etherification. All polymer conjugates were fully characterized in vitro and the pharmacokinetic profile of selected conjugates was characterized in rats. Buffer and plasma stability of conjugates was dependent on polymer hydrophobicity, aggregation behavior and PEG corona, with MeO-PEG-PLAO-Succ-RSV (2 kDa) showing a 3h half-life in rat plasma in vitro. Polymer conjugates irrespective of linker chemistry protected resveratrol against metabolism in vitro. MeO-PEG-PLAO-Succ-RSV (2 kDa), Resveratrol-PEG ether (2 and 5 kDa) displayed improved pharmacokinetic profiles with significantly higher plasma area under curve (AUC), slower clearance and smaller volume of distribution, compared to resveratrol.

## Introduction

Resveratrol is a naturally occurring polyphenolic phytochemical with beneficial biological activities such as anti-inflammatory, anticancer, antioxidant, and cardio protection [1–3]. Diverse biological activities of resveratrol are due to its intervention in multiple inflammatory pathways [2]. More recently, resveratrol has been confirmed to be an allosteric activator of SIRT1 [4], an enzyme that belongs to the sirtuin family of nicotinamide adenine dinucleotide (NAD+)-dependent deacetylases. SIRTI1 is known to be involved in many fundamental cellular processes including gene silencing, DNA repair, and metabolic regulation, which are all related to aging. Price and his colleagues [4] showed that activation of SIRT1 by resveratrol increased mitochondrial biogenesis and improved mitochondrial function. Hence resveratrol is also considered to be a potential therapeutic candidate for treating age-related disorders and increasing lifespan of individuals. It is not surprising that resveratrol is widely used as a nutritional supplement for various health benefits. However the health benefits of resveratrol are equivocal because of its poor pharmacokinetic profile [5–7].

The pharmacokinetic profile of resveratrol after oral administration in humans was described as high absorption but very low bioavailability due to extensive first-pass metabolism and short elimination half-life [8]. This rapid metabolism pattern has been seen in other species including mouse, rat, pig and dog [9–12]. After intravenous administration to rats, the elimination half-life of resveratrol (plasma) was 0.13 h [6]. Since resveratrol is polyphenolic in nature, hydroxyl groups of resveratrol were found to be targets for metabolism via conjugation and accordingly various conjugated sulfate and glucuronide metabolites of resveratrol were detected in the plasma; however, resveratrol-3-O-sulfate and resveratrol-3-O-glucuronide were found to be the major metabolites [13]. Although the understanding of biological activities of these metabolites remains elusive, the shorter plasma residence of resveratrol has been reported to be inadequate to elicit biological activities *in vivo* [14].

Resveratrol was found to be effective in certain *in vivo* animal models where the native un-metabolized resveratrol is accessible at the site of action. Resveratrol has shown a significant chemopreventive effect in azoxymethane induced carcinogenesis in a rat colon cancer model when given orally in drinking water [14]. Similarly resveratrol was also found to be an effective chemopreventive agent in a mouse skin cancer model with 7, 12-dimethylbenzanthracene (DMBA) as an initiator, and 12-O-tetradecanoylphorbol-13-acetate [TPA] as tumour promoter. The number of tumours per mouse was significantly reduced compared to controls [15]. Such effects were attributed to the presence of the native resveratrol at the site of action, as the resveratrol did not pass through the systemic circulation to reach the site of action. However there was a reported discrepancy between *in vitro* and *in vivo* anti-leukemic effects of resveratrol. Despite showing a strong anti-leukaemic effect in the *in vitro* studies, resveratrol failed to show a promising anti-leukaemic effect *in vivo* and did not protect mice injected with leukaemia cells [16]. The inability of resveratrol to arrest leukaemia cell differentiation in the mice was attributed to its rapid metabolism, and was in stark contrast to *in vitro* observations. In a study conducted by Wenzel et al. to evaluate the chemopreventive and antioxidant activities of resveratrol, the researchers concluded that chemopreventive parameters such as levels of certain phase-I and phase-II metabolizing enzymes, and total antioxidant activity were unaltered in rats after 8 weeks of resveratrol treatment at various doses given with food (particularly at 300 mg high dose) [7]. The lack of native resveratrol in the body was considered to be responsible for the inactivity of reseveratrol.

Polymer conjugation is a strategy used to improve the circulation time of peptides/proteins and small drug molecules. Studies have shown that conjugation with polymers significantly reduced the elimination rate of peptides/proteins by proteolytic degradation, as well as immunogenic and antigenic responses, thereby prolonging circulation times [17–18]. Polymer conjugation approaches have successfully improved safety, efficacy and pharmacokinetic profiles of small molecule drugs such as doxorubicin, norfloxacine, campothecine and paclitaxel [17–23]. However most of these drugs are metabolically stable and the feasibility of employing polymer conjugation to protect small molecules such as resveratrol against metabolism needs to be fully explored. Structure-activity relationship studies of resveratrol revealed that hydroxyl groups in the structure are essential for its biological activity [24–25]. Hence resveratrol delivery approaches based on polymeric conjugation should not permanently block hydroxyl groups and, even if permanent conjugation is considered, only selected hydroxyl groups should be modified with appropriate polymers and with minimum impact on the biological activity of resveratrol.

The major limitation in developing polymeric conjugates of small molecules is the absence of functional groups amenable for conjugation and the reported poor stability of conjugates in plasma due to their hydrolysis by plasma esterases and carboxypeptidases. This is also a major reason that polymeric conjugates were largely exploited as prodrugs for improving solubility of small molecules such as paclitaxel [23].

The objective of our investigation was to evaluate various polymer conjugation technologies for stabilizing resveratrol against metabolism and consequent impact on resveratrol’s pharmacokinetics profile and in turn its biological effects *in vivo*. Resveratrol polymer conjugates were prepared using ester and ether linker chemistry with different molecular weight of polymers. In this study, efforts were made to understand the effect of the physicochemical properties of polymers and chemistry of linkers on the stability of conjugates in buffer and plasma. Finally the selected polymer conjugates were also evaluated for *in vitro* antioxidant activity, metabolism and pharmacokinetic profile in Wistar rats.

## Materials and Methods

### Materials

*trans*-Resveratrol (99%: DND Pharma-Tech Co., Inc, Shanghai, China) was used throughout the study as this isomer was reported be more active than the *cis*-isomer. α-Methoxy-ω-carboxylic acid poly(ethylene glycol) succinylamide acid (MeO-PEGN-SuccOH) of molecular weight (MW) 2 kDa and 20 kDa, methoxypoly(ethylene glycol)bromide (MeO-PEG-CH_2_CH_2_Br), 2 kDa and 5 kDa were obtained from IRIS Biotech GmbH (Germany). Methoxypoly(ethylene glycol) (MeO-PEG-OH, MW 2 kDa), methoxypoly(ethylene glycol)-co-polylactide (MeO-PEG-PLA-OH, Mw 2 kDa) and succinic anhydride were purchased from Sigma-Aldrich Ltd. (Sydney, Australia). MeO-PEG-PLA-OH 6.6KDa was purchased from Akina Inc (Indiana, USA). N,N'-Diisopropylcarbodiimide (DIC) and 1-ethyl-3-(3-dimethylaminopropyl)carbodiimide hydrochloride (EDC.HCl) were obtained from GL Biochem (Shanghai, China). Rat liver microsomes (20mg/mL protein) were obtained from Invitrogen (Mulgrave, Australia). UDP-glucuronosyltransferases (UGT A) and UGT B solutions were purchased from BD Bioscience (San Jose, CA, USA). Dimethyl yellow was obtained from Sigma. 0.1 M acetate buffer pH 4.5 and 0.1 M phosphate buffer pH 7.4 were prepared in-house. All remaining chemicals such as potassium carbonate, dichloromethane (DCM), pyridine, toluene and diethyl ether were of analytical grade and used without any further purification. Male Wistar rats (180–220g) and rat plasma were obtained from the Animal Resource Centre, Murdoch, Western Australia. The animal studies were approved by the Animal Ethics Committee of Curtin University and were performed according to the Australian Code of Practice for the care and use of animals for scientific purposes.

#### Synthesis of functionalized polymer and Resveratrol-Polymer conjugates

##### Synthesis of MeO-PEGO-SuccOH

MeO-PEG-OH (2 kDa, 5g, *ca* 2.5 mmol) was dissolved in toluene (50 mL) and succinic anhydride (1.2g, 12 mmol) was added to the solution. The resulting solution was heated at 90°C for 4 h. The solution was concentrated to 10 mL *in vacuo* then cooled to room temperature. Diethyl ether (150 mL) was added to precipitate the MeO-PEGO-SuccOH. The precipitate was collected by filtration and air-dried.

##### Synthesis of MeO-PEG-PLAO-SuccOH

MeO-PEG-PLA-OH, (Mw 6.6kDa, 1 g, ca 0.15 mmol) was dissolved in toluene (10 mL) and an excess of succinic anhydride (0.2 g, 2 mmol) was added to mixture. The reaction mixture was refluxed at 90°C for 4 hours in a round bottom flask fitted with a condenser. At the end of the reaction, the mixture was cooled to room temperature and diethyl ether (100 mL) was added to precipitate the MeO-PEG-PLAO-SuccOH. The precipitate was isolated by filtration and characterized by NMR. A similar process was used to prepare MeO-PEG-PLAO-SuccOH Mw 2 kDa.

##### Synthesis of Resveratrol-PEG conjugates

DIC (0.1 g, 0.79 mmol) was added to a solution of MeO-PEGN-SuccOH (Mw 2 kDa, 1 g, *ca*. 0.5 mmol) and excess resveratrol (0.25 g, 1.09 mmol) in pyridine (2 mL) and the resulting solution was stirred in darkness, at room temperature for 24 h. The solution was filtered (0.44 μm filter) and the filtrate was diluted with diethyl ether (150 mL). The resulting precipitate was then washed with a solution of ethyl acetate/diethyl ether (20 mL/80 mL), followed by a solution of ethyl acetate/diethyl ether (50 mL/50 mL), and then dried *in vacuo* to afford an off-white material (300 mg, 30% yield). A similar procedure was followed for preparation of resveratrol conjugates with MeO-PEGN-SuccOH 20 kDa and MeO-PEGO-SuccOH 2 kDa.

##### Synthesis of Resveratrol-PLA-PEG conjugates

MeO-PEG-PLAO-SuccOH, (Mw 6.6kDa, 1 g, ca 0.15 mmol) was dissolved in pyridine (5.0 mL). The excess of resveratrol (0.15g, 0.65 mmol) and EDC.HCl (0.15 g, 0.78 mmol) were added to the mixture. The reaction mixture was stirred at room temperature for 48 hours under dark conditions. At the end of the reaction, the mixture was added to diethyl ether (150 mL) and the product was isolated by filtration. The product was dissolved in DCM (50 mL) and was extracted with 0.1 M HCl (50 mL) twice to remove any unreacted EDC.HCl and its urea by-product. Finally DCM layer was evaporated *in vacuo* to obtain an off-white powder (500 mg, 50% yield). The MeO-PEG-PLAO-Succ-Resveratrol, Mw 2 kDa was also prepared with the same procedure.

##### Synthesis of Resveratrol-PEG ethers

MeO-PEG-CH_2_-CH_2_-Br, (Mw 2 kDa, 1 g, 0.5 mmol) and resveratrol (0.15 g, 0.657 mmol) were dissolved in of DMF (5 mL). K_2_CO_3_ (0.25 g, 1.81 mmol) was added to solution and mixture was stirred at room temperature for 24 hours in dark. At the end of the reaction, the mixture was added to DCM (50 mL), solution was filtered to remove potassium bromide produced in the reaction and also unreacted K_2_CO_3_. The filtrate was extracted with 0.1 M HCl solution (50 mL) to remove traces of KBr and K_2_CO_3_. The extraction process was repeated two more times and DCM layer was evaporated *in vacuo* to yield an off white to slightly brown product (500 mg, 50%). The resveratrol-PEG ether with MeO-PEG-Br Mw 5 kDa was also prepared with the same procedure.

#### Characterization of resveratrol-polymer conjugates

##### Characterization of polymer conjugates and their precursors by ^1^H NMR

Dried powder samples of reaction products obtained from Section 2.2 to 2.6 were analysed by ^1^H NMR to elucidate the chemical structures of compounds/mixtures. All ^1^H NMR analyses were performed on a Bruker AVN400 spectrometer (400.1 MHz for ^1^H) with spectra referenced to solvent signals.

##### Characterization of Polymer conjugates by LC-MS

The LC-MS analysis of polymer conjugates was performed to confirm formation of conjugates, extent of drug conjugation to polymer and extent of polymer functionalization. All the LC-MS experiments were performed on a Shimadzu LC-MS 2020 system (Kyoto, Japan) as per our previously published method [26]. The system consists of a binary pump 20AD, auto injector SIL 20 AC HT, column oven CTO 20A, PDA detector SPD M 20A, mass analyser MS2020 and communication unit CBM 20A. Data were acquired, processed and analysed using chromatographic software LABSOLUTIONS. Chromatographic elution was performed using a linear gradient with water and acetonitrile as the mobile phase system. The percentage of acetonitrile in the mobile phase was: at 1 min 40%, 4 min 95% and 8 min 40%. A Zorbax SBC-18 column was employed for separation at column oven temperature of 30°C. The mass detector parameters were set as follows: DL temperature 300°C, interface temperature 350°C and the heating block temperature was maintained at 220°C, with drying gas nitrogen flow at 10 L/min. The selected ion monitoring (SIM) was carried out with positive ions 700, 720, 730, 900, 1000 for PEG fragments and 227 for resveratrol [26].

##### Polymeric micelles preparation and characterization

A solution of 40 mg of MeO-PEG-PLAO-Succ-RSV conjugate in acetonitrile (2 mL) was prepared. The stock solution was diluted with water (4 mL). The acetonitrile was removed by bubbling inert nitrogen gas at room temperature in the dark. The final volume of micellar/nanoparticle dispersion was adjusted to the required volume with water. For pharmacokinetic studies, final concentration of conjugate in micelle dispersion was 10 mg/mL and for critical micellear concentration measurement, the dispersion was diluted accordingly to obtain a series concentration of conjugate in solution below and above the CMC. The micelles of MeO-PEGO-Succ-RSV were formed spontaneously after dispersing in water.

##### Measurement of micelles size

Particle size and size distribution of the resveratrol polymer conjugate micelles were measured by a Zetasizer (3000HS, Malvern Instruments, UK), using a quartz cell with the automatic mode at 25°C and a detection angle of 90°. Mean diameters (Z-average) were obtained after 10 repeated measurements.

##### Determination of critical micellar concentration (CMC) values for resveratrol polymer conjugates

CMCs for various micelles-forming polymer conjugates of resveratrol were determined by dye solubilization technique using dimethyl yellow as UV probe (27). Both absorbances at fixed wavelength and shift in absorbance maxima of dimethyl yellow were monitored as the function of concentration of polymer conjugate. A concentrated solution of dimethyl yellow (10 mg/mL) was prepared in acetone. The initial concentrated MeO-PEG-PLAO-Succ-RSV micelle solutions were prepared by diluting 125 μL of polymer conjugate stock solution (20 mg/mL) in acetonitrile to 100 mL with water as per previous section. After removing acetonitrile, this solution was further diluted with water to obtain a series of solutions with various concentrations of polymer conjugates (1–25 mg/L). The MeO-PEGO-Succ-RSV solutions were prepared by dissolving 40 mg of conjugate in 10 mL of watewith further serial dilution to produce solutions of concentrations from 0.005 to 0.4% (50 to 4000 mg/L). These polymer conjugate solutions were added separately to vials containing dimethyl yellow and sonicated for 10 minutes. The solutions were stored in the dark for 3 h to equilibrate. These solutions were then scanned for max using a UV-Visible spectrophotometer between 300 and 510 nm. The change in wavelength of maximum absorption and absorbance at fixed wavelength 441nm were noted against polymer conjugate concentration. The CMC values were determined from a plot of the log of concentration (log C) of polymer conjugates versus max (or absorbance at 441 nm).

##### Micelle morphology by atomic force microscopy (AFM) and field emission scanning electron microscopy (FESEM)

Atomic Force Microscopy was used to visualise the conjugate samples. All measurements were taken in Tapping Mode operation with a Digital Instruments Dimension 3000 SPM system (Veeco, Santa Barbara, USA). Standard silicon Tapping Mode probes type NCH (NanoWorld, Switzerland) with resonant frequency 330 kHz, spring constant 42 N/m and tip radius < 8 nm. The AFM samples were prepared by drop casting the micelle solution onto a freshly cleaved mica substrate. The sample was placed in a desiccator attached to a vacuum pump to remove excess solvent.

Morphological evaluation of micelles was performed using FESEM instrument, Zeiss Neon 40 ESB (Oberkocken, Germany). For FESEM, one drop of micelle solution was mounted on a carbon-coated stub. Excess sample was removed carefully and the stud was dried in air. A Polaron E5100 sputter-coater was used to sputter coat the samples with chromium. Samples were then examined under the FESEM instrument with an accelerating voltage of 1 kV.

##### Stability of resveratrol-polymer conjugates in physiological relevant buffers

The stability of resveratrol polymer conjugate was evaluated at different pH buffers 4.5 (acetate buffer 0.1M) and 7.4 (phosphate buffer 0.1M) to mimic lysozomal and blood pH. The resveratrol polymer conjugates of different Mw and linkers were incubated in the two buffers at a concentration of 10 mg/mL at 37°C, the samples were withdrawn at 0, 1, 2, 4, 6 and 24 hours and were diluted with water/acetonitrile (50:50) and were analysed by HPLC [28] using an Agilent Technologies chromatographic system 1100 series (Santa Clara, CA, USA) with dual fluorescence and PDA detectors. Briefly, reversed phase chromatographic separation was employed for analysis with xxx column. The HPLC gradient system consisted of solvent A, buffer pH 7.0 with triethyl amine-formic acid (0.05 and 0.2% v/v in water) and solvent B, methanol. A 30 min linear gradient was programmed as follows: 0 min 5% B, 4 min 20% B, 7 min 20% B, 16 min 55% B, 18 min 55% B, 18.5 min 95% B, 23 min 95% B, 24 min 5% B. The flow rate was maintained at 1 mL/min and the injection volume was 50 μL. The HPLC analysis was performed at ambient temperature. Altech Apollo C18 150 X 4.6 mm, 5μ column was employed for seperation. This HPLC method was validated to precisely separate and quantify the *trans*-resveratrol in the analyte mixture. There was at least two min difference in retention times for the *trans* and *cis* isomers of resveratrol. The UV spectrum of the *trans* isomer is very different from the *cis* isomer, thus any change to the *trans* isomer could have been detected. Although quantification was performed using the fluorescence detector at an excitation wavelength of 320nm and emission at 400 nm, simultaneous UV chromatograms were also recorded for specificity and peak purity purposes.

A similar procedure was used to study the stability of MeO-PEG-PLAO-Succ-RSV in buffers; micelles were prepared as per section 2.8 and diluted 1:5 with buffer solutions and incubated at 37°C. All samples were protected against light during the experiments.

##### Stability of resveratrol-polymer conjugates in rat plasma

The stability of various resveratrol polymer conjugates in rat plasma was tested in order to assess the stability of the ester linkages towards plasma esterases. The **MeO-PEGN-Succ-RSV**, 2 kDa, 20 kDa and **MeO-PEGO-Succ-RSV** 2 kDa (MW quoted in the paper is the size of polymer only. Mw for RSV is 228 Da) at the 10 mg/mL were incubated in plasma at 37°C. The micelles of **MEO-PEG-PLAO-Succ-RSV** 2 kDa and 6.6 kDa were prepared and, finally the micelles were mixed with plasma and incubated at 37°C. Aliquots of plasma (0.1 mL) were withdrawn at regular intervals and mixed with acetonitrile (0.9 mL). The mixtures were centrifuged at 10,000 rpm for 5 minutes. The supernatant (0.25 mL) was diluted with water (0.25 mL) and analysed by HPLC [28]. All samples were protected against light during the experiments.

##### In vitro preliminary metabolism studies of resveratrol and its polymer conjugates in rat liver microsomes

Resveratrol has been reported to undergo only phase II metabolism, such as glucuronidation and sulfation (6–8), therefore the metabolism studies with rat liver microsomes were conducted in the presence of uridine diphosphoglucuronic acid (UDPA). The stock solutions of resveratrol (0.13 mM) and polymer conjugates (0.13 mM resveratrol equivalents) were prepared in 10% v/v DMSO in water. The rat liver microsomes, (UDP-glucuronosyltransferases (UGT) solution A (contains UDPA) and UGT solution B were thawed at 37°C. The 200 μl of UGT solution B, 80 μl of UGT solution A and 660 μl of water were mixed, and 10 μl of the test substance stock solution (resveratrol or conjugates) was added to the mixture. The mixture was kept at 37°C. The reaction was initiated by adding 50 μl of microsomes (1mg protein). Samples (250μl) from reaction mixtures were withdrawn at 0, 10, 20, 30, 40 and 60 min and mixed with equal volumes of cold acetonitrile. The mixtures were centrifuged at 7000 × g for 10 min and supernatants were collected and analyzed by HPLC. Incubations of test substances in reaction mixtures without microsomes were used as a control. All samples were protected against light during the experiments.

##### In vitro antioxidant assay of resveratrol and its polymer conjugates

The free radical scavenging effects of resveratrol and its polymer conjugates were evaluated using 1,1-diphenyl-2-picrylhydrazyl (DPPH) inhibition assay. Both DPPH solution (2 x 10^-4^M) and a series of solutions of resveratrol and resveratrol-PEG ester conjugates were prepared in methanol. The DPPH solution of 1mL was mixed with 1 mL of resveratrol solution. Final concentration of DPPH was maintained at 1 x 10^-4^M. The procedure was repeated for other resveratrol solutions of various concentrations and also for resveratrol-PEG ester solutions. The concentration of resveratrol in testing solutions ranged from 21–877 μMol/L and 210 to 8770 μMol/L for polymer conjugates. After 30 min incubation at room temperature, the absorbance of these solutions was determined at 518 nm using a Hewlett Packard 8452A, UV-VIS spectrophotometer (Waldbronn, Germany). The DPPH concentration remaining in solution was calculated by comparing with control, a standard solution of DPPH without any resveratrol or its conjugate. The IC 50 values were determined from the plot of % DPPH remaining against concentration of RSV and its equivalent conjugates.

##### Preliminary pharmacokinetic study of resveratrol and its polymer conjugates in Wistar rats with IV injection

Pharmacokinetic studies were conducted with male Wistar rats. Rats were acclimatized and housed at 22°C under 12h light/dark cycle with free access to sterilized commercial food pellets and water in the animal research facility (Curtin University) for at least 3 days prior to experiments. All animal experiments were approved by the Animal Ethics Committee of Curtin University (Approval no: R37–10 Amendment Approval No: A47–10 and A56–10) and were performed according to the Australian Code of Practice for the care and use of animals for scientific purposes. In order to minimize pain and suffering, the dosing and bleeding procedures were conducted under anesthesia (isoflurane at 5% for the induction and 2% for the maintenance, in combination with 1.5% oxygen). At the end of the study, rats were scarified by anesthetic euthanasia using 5% isofluorane with 1.5% oxygen. Each treatment group was assigned 5–6 rats and received one of the following treatments: resveratrol solution, **MeO-PEG-PLA-Succ-RSV** 2 kDa and 6.6 KDa, RSV-PEG ethers 2 kDa and 5 kDa via intravenous injection into the tail vein. A resveratrol solution (6 mg/mL), prepared in 20% hydroxyl-propyl-β-cyclodextrin, was dosed to Wistar rats at 10 mg/kg body weight. Polymeric micelles of the **MeO-PEG-PLA-Succ-RSV** 2 kDa and 6.6 kDa at a concentration of 10 mg/mL of conjugate were prepared as per section 2.8 and dosed to rats at a dose of 2 mg/kg of resveratrol equivalent. The 20 mg/mL solution of RSV-PEG ethers 2 kDa and 5 kDa was prepared by dissolving ether conjugates in water and dosed at 2 mg/kg of resveratrol equivalent. Blood (300 μL) was collected from the tail vein at 0.08, 0.5, 1, 2 and 4 hours into EDTA coated tubes. The blood samples were immediately stored in ice and protected from light until centrifuged (12000 × g at 4°C for 10 min). The separated plasma (100 μL) was mixed with an equal volume of acetonitrile and centrifuged (12000 × g at 4°C for 12 min). The supernatant was collected and the concentrations of resveratrol and resveratrol equivalent (polymer conjugates) were determined using a validated HPLC method [28]. Metabolites were identified using LC-MS/MS (Applied Biosystems, 4000 QTRAP, Toronto, Canada) [26].

##### Statistical and pharmacokinetic analyses

Data analysis and representation (mean ± SD) were performed with SigmaPlot version 12 (Systat Software, Inc., Chicago, IL). Pharmacokinetic analysis was performed using Kinetica Version 5.0 (Thermo Fisher Scientific, Inc., Waltham, MA). Non compartmental analysis of the plasma concentration-time data was used to estimate the area under the curve (AUC_0-∞_; log-linear trapezoidal method), elimination half-life (t_½_), apparent clearance (CL; Dose÷AUC_0-∞_), mean residence time (MRT) and volume of distribution (V_ss_; CL×MRT). Statistical analysis of pharmacokinetic parameters was performed using unpaired t-test with Graphpad Prism software (version 6.05). Pharmacokinetic parameters obtained for each group were analyzed against the resveratrol group and p values were reported.

## Results and Discussion

### Synthesis of succinic acid terminated polymers for resveratrol conjugation

The esterification of MeO-PEG-OH and MeO-PEG-PLA-OH with succinic anhydride was chosen for coupling of polymers to hydroxyl groups of resveratrol. The esterification was achieved by ring opening reaction mediated by heat in an inert solvent toluene. This was a modified method adopted from Kuang et al [29]. The reaction worked well for both MeO-PEG-OH and MeO-PEG-PLA-OH irrespective of their polymer chain length and composition. The success of esterification was confirmed by the presence of two triplets at 2.3 and 2.4 ppm in NMR, which are characteristic triplet peaks of ethylene protons of succinic acid. In addition, the esterification of MeO-PEG-OH was supported by LC-MS analysis [26] as there was absence of MeO-PEG-OH peak in LC-MS chromatogram but MeO-PEGO-SuccOH peak was detected, indicating 100% conversion of MeO-PEG-OH to MeO-PEGO-SuccOH.

### Synthesis and characterization of resveratrol-Polymer conjugates

**Fig 1:**
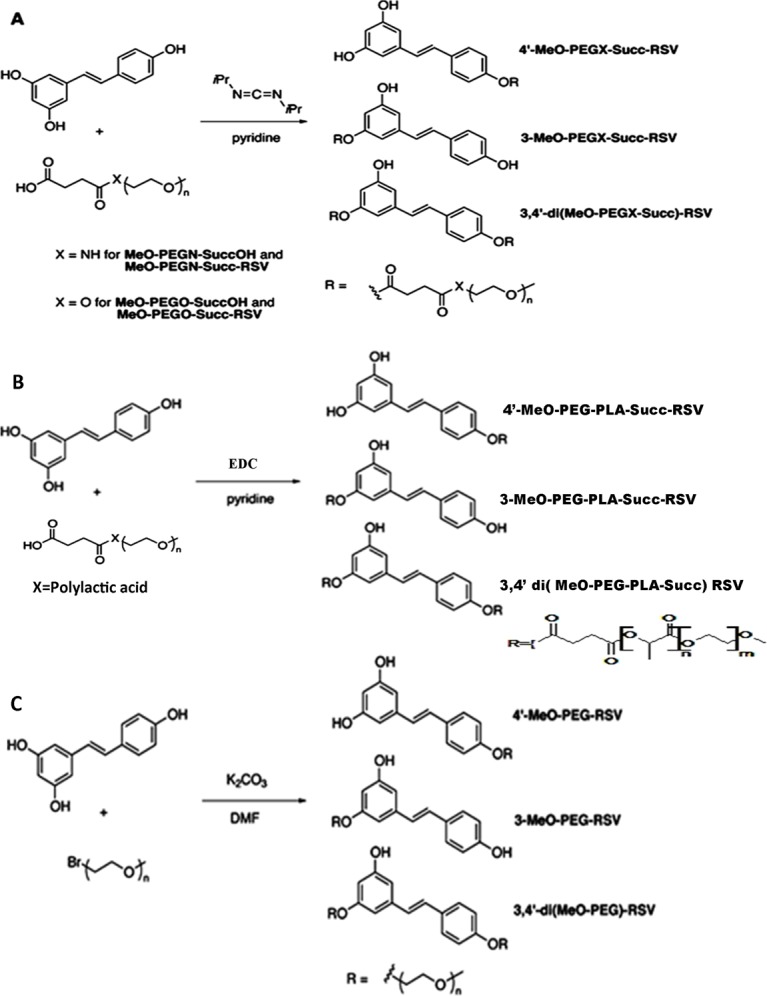
Synthesis of (A) resveratrol-PEG ester conjugates, (B) MeO-PEG-PLAO-Succ-RSV conjugates and (C) resveratrol-PEG ether conjugates.

**Fig 2:**
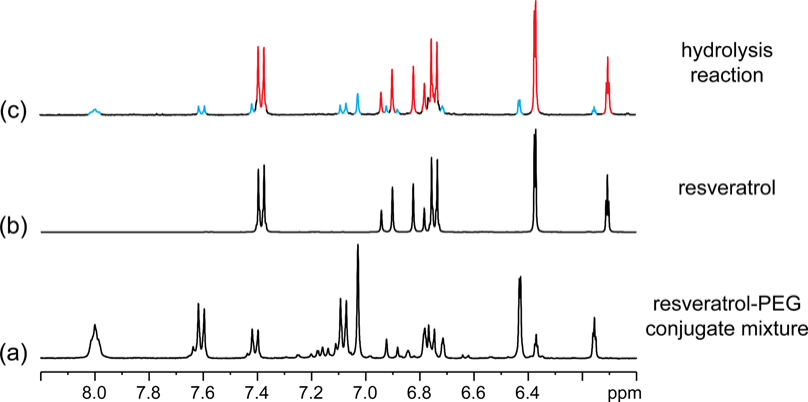
The down field region of ^1^H NMR spectra (400 MHz) for: (a) a solution of the MeO-PEGN-Succ-RSV (2 kDa) conjugate mixture in *d*_6_-DMSO; (b) a solution of resveratrol in *d*_6_-DMSO; and (c) the solution from (a) after treatment with water and heat (90°C) for *ca*. 1 week). In (c) the signals for free resveratrol are highlighted in red and those for the remaining resveratrol-PEG conjugates are highlighted in blue. Full width versions of these spectra are provided in S1 File, along with a comparison with the MeO-PEG-Succ-OH starting material.

Resveratrol-PEG conjugates were synthesized by carbodiimide coupling, using a modified method reported by Greenwald et al. for the PEGylation of 6-hydroxyquinoline [30]. The reaction was conducted with excess resveratrol and **MeO-PEGN-SuccOH** (2 kDa) in the presence of diisopropylcarbodiimide and pyridine, which afforded a mixture of resveratrol-PEG conjugates (Fig. 1A), with a final yield of 30%. The low yield was due to the loss of compound(s) during repeated washing with diethyl ether/dichloromethane to remove un-reacted resveratrol from the mixture. The NMR analysis of the product (s) suggested that the obtained mixture included two mono-PEGylated resveratol conjugates **4'-MeO-PEGN-Succ-RSV** (*ca*. 50%) and **3-MeO-PEGN-Succ-RSV** (*ca*. 35%) as the major products, and what we believe is a disubstituted derivative **3,4'-di(MeO-PEGN-Succ)-RSV** as a minor product. The identification of the mixture, and the substitution pattern, was based on the ^1^H NMR spectra obtained for the resveratrol-PEG conjugate mixture (see Fig. 2 and Figs. A and B in S1 File). The ^1^H NMR spectra revealed that the conjugate mixture contained no free resveratrol, as indicated by the absence of a signal at *ca*. 6.1 ppm in Fig. 2(a). This signal, observed in Fig. 2(b), is due to H-4 of free resveratrol (see Fig. B in S1 File for assigned spectra). As a control, a solution of resveratrol and **MeO-PEGN-SuccOH** in *d*_6_-DMSO was analysed by ^1^H NMR spectroscopy. The chemical shifts of the signals for resveratrol in the mixture were identical to that shown in Fig. 2(b) for resveratrol in *d*_6_-DMSO. The ^1^H NMR spectra for the resveratrol-PEG conjugate mixture also indicated that no free **MeO-PEGN-SuccOH** remained in the material. The signals corresponding to the succinyl moiety in **MeO-PEGN-SuccOH** were absent in the spectra for the resveratrol-PEG conjugate mixture (see Fig. A in S1 File). The assignment of signals corresponding to **3-MeO-PEGN-Succ-RSV** and **4'-MeO-PEGN-Succ-RSV** in Fig. B in S1 File was based on a comparison with the spectra for resveratrol and 2D NMR spectra (COSY). Additional minor signals remain unassigned in Fig. B in S1 File. We believe these signals are due to a third product, **3,4'-di(MeO-PEGN-Succ)-RSV** which was formed as a minor component. To confirm this assumption a sample of the resveratrol-PEG conjugate mixture, formed in the synthesis of **MeO-PEGN-Succ-RSV**, in *d*_6_-DMSO was treated with water and then heated at 90°C for *ca*. 1 week, to hydrolyse the phenolic ester linkages. The ^1^H NMR spectrum for the sample, shown in Fig. 2(c) (and Fig. A in S1 File), revealed the majority of the material had hydrolysed to afford free resveratrol. The remaining signals shown in Fig. 2(c) can be attributed to residual **3-MeO-PEGN-Succ-RSV** and **4'-MeO-PEGN-Succ-RSV**. Interestingly, the hydrolysis of the resveratrol-PEG conjugate did not produce MeO-PEGN-SuccOH as the other hydrolysis product, though this may have formed as an intermediate. The ^1^H NMR spectra for the solution after the hydrolysis reaction correlate with the formation of a succinimide-terminated PEG chain. Fig. A(d) in S1 File displays a singlet at *ca*. 2.6 ppm which is consistent with the succinimidyl ring methylene protons, and the absence of the succinyl ester amide protons observed for MeO-PEGN-SuccOH (*c*.*f*. Figs. A(a) and A(d) in S1 File). The formation of a succinimide ring is consistent with literature observations for the hydrolysis of the succinyl ester amide moiety [31–33]. Integration of the signals for resveratrol and the succinimide-terminated PEG in Fig. A(d) in S1 File indicated that there was more succinimide-terminated PEG than free resveratrol, which supports our assumption that the minor component of the resveratrol-PEG conjugate was the disubstituted product **3,4'-di(MeO-PEGN-Succ)-RSV.** The full blown ^1^H NMR spectra of MeO-PEGN-Succ-RSV, MeO-PEGN-Succ-OH, MeO-PEGN-Succ-RSV after heat treatment are shown in Figs. C, D, E in S1 File respectively.

The ^1^H NMR spectra for the other PEG ester conjugates of resveratrol showed that the conjugation pattern was similar to that of MeO-PEGN-Succ-RSV (2 kDa). However, the extent of PEG conjugation to resveratrol varied between PEGs of different MW and acid functional groups. The conjugation was almost *ca* 100% for MeO-PEGN-SuccOH 2 kDa, *ca*. 70% and 50% respectively for MeO-PEGO-SuccOH 2 kDa and MeO-PEGN-SuccOH 20 kDa, However, in the synthesis of MeO-PEGO-Succ-RSV 2 kDa and MeO-PEGN-Succ-RSV 20 kDa the isolated products contained a significant amount of an N-acyl urea as a by-product, which is formed by isomerisation of the O-acylisourea"active ester" that results from the reaction of the carbodiimide and the PEG acid [34–35]. This indicated the reaction of the active ester and resveratrol was slow and incomplete.

For synthesis of MeO-PEG-PLAO-Succ-RSV, the successful conjugation was achieved using EDC as a coupling agent and pyridine as the base (Fig. 1B). The ^1^H NMR analysis of MeO-PEG-PLAO-Succ-RSV 2 kDa indicated that the product was a mixture of conjugates **4'-MeO-PEG-PLAO-Succ-RSV**(*ca*. 50%) and **3-MeO-PEG-PLAO-Succ-RSV** (*ca*. 35%) as the major products, and a disubstituted derivative, presumably **3,4'-di (MeO-PEG-PLAO-Succ)-RSV**, as a minor product. The extent of conjugation was almost 80%. The substitution pattern for MeO-PEG-PLAO-Succ-RSV 6.6 kDa was found similar to MeO-PEG-PLAO-Succ-RSV 2 kDa, but contained certain amount of free resveratrol and unreacted polymer (<1%). The free resveratrol, however, can be separated by HPLC from conjugates and did not interfere with the study.

The synthesis of resveratrol-PEG ethers was achieved using PEG-Br in the presence of K_2_CO_3_ in DMF (Fig. 1C). Initially the synthesis of the PEG ether was attempted with K_2_CO_3_ and acetone [36] at reflux (60°C) but no product was isolated. The combination of K_2_CO_3_ and DMF yielded favorable results and conjugation was found to be dependent on amount of K_2_CO_3_ in the reaction mixture. The reaction was monitored by HPLC, which indicated that conjugation was highest when the concentration of K_2_CO_3_ in the reaction mixture was 1.8 mM. The ^1^H NMR analysis of resveratrol-PEG ether 2 kDa revealed that **4'-PEG-RSV** was a major product (*ca*. 60%), along with **3-PEG-RSV**(*ca*. 30%) and a minor **3,4'-di(PEG)-RSV** (*ca*. 10–15%). The extent of conjugation was approximately 80%. The pattern of substitution and conjugation chemistry was similar for the resveratral-PEG ether 5 kDa [37]. The various resveratrol-polymer conjugates synthesized in the study are listed in Table 1, along with their physicochemical properties.

**Table 1:** Physicochemical properties and stability data of Resveratrol-polymer conjugates.

RSV-polymer conjugates	PLA Mw	PEG MW (Daltons)	% Conjugate Remaining at 24 hr pH 4.5	% Conjugate Remaining at 24 hr pH 7.4	Plasma t_1/2_	CMC mg/L	CMC Moles/L
MeO-PEG-PLAO-Succ-RSV 2 kDa	1000	750	99.2	104	3 hours	4	0.002
MeO-PEG-PLAO-Succ-RSV 6.6 kDa	4600	2000	78.4	78.7	5 minutes	8	0.0012
MeO-PEGO-Succ-RSV 2 kDa	N/A	2000	89.1	64.2	<5 minutes	500	0.25
MeO-PEGN-Succ-RSV 2 kDa	N/A	2000	86.4	7.6	<5 minutes	N/A	N/A
MeO-PEGN-Succ-RSV20 kDa	N/A	20000	88.1	9.3	<5 minutes	N/A	N/A
MeO-PEG-RSV ether 2 kDa	N/A	2000	100	100	No hydrolysis stable	N/A	N/A
MeO-PEG-RSV ether 5 kDa	N/A	5000	100	100	No hydrolysis stable	N/A	N/A

N/A: not applicable as conjugates are soluble.

### Characterization of resveratrol-polymer conjugates

**Fig 3:**
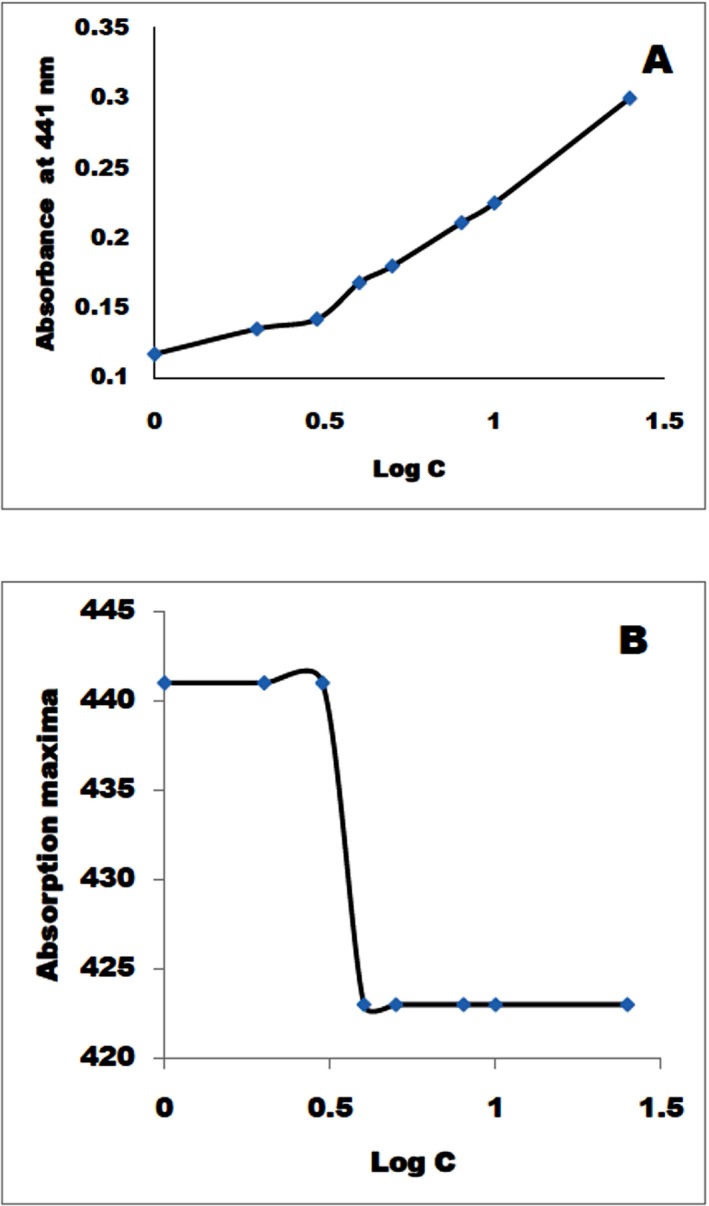
Determination of CMC for MeO-PEG-PLAO-Succ-RSV 2kDa. A: plot of log (Concentration mg/L) vs Absorbance for dimethyl yellow at 441. B: plot of log (Concentration mg/L) vs Absorption maximum (nm) for dimethyl yellow. Mw quoted is for polymer only. Mw for RSV is 228 Da.

**Micelles characterization.** The resveratrol conjugates consisting of PLA-PEG were formulated into micelles then characterized both *in vitro* and *in vivo*. Detailed micelles characterization was performed to correlate various physicochemical properties to *in vitro* and *in vivo* stability of micelles. It was interesting to note that **MeO-PEGO-Succ-RSV** 2 kDa self-aggregated into polymeric micelles, as the solution turned turbid during the *in vitro* buffer stability study. The self-aggregation of **MeO-PEGO-Succ-RSV** into micelles presumably occurred because of the hydrophobicity of resveratrol. The solubilizing power of PEG MW 2 kDa was not sufficient to counter the hydrophobicity of resveratrol, hence self-assembly into micelles was favored. Veronese *et al* [38] reported similar findings with doxorubicin-PEG (DOX-PEG) conjugates with a tetra-peptide linker (GFLG). The authors found that the tendency to aggregate into polymeric micelles was dependent on the molecular architecture and MW of the polymer. The DOX-GFLG-PEG with PEG5000 aggregated more readily than with PEG10000. This was attributed to greater solubilisation effect of PEG10000. In stark contrast to MeO-PEGO-Succ-RSV 2kD, the **MeO-PEGN-Succ-RSV** 2 kDa was soluble and formed a clear solution with no cloudiness even at a high concentration (10 mg/mL). The CMC for **MeO-PEG-PLA-Succ-RSV** 2 kDa, **MeO-PEG-PLA-Succ-RSV** 6.6 kDa and **MeO-PEGO-Succ-RSV** 2 kDa were found to be 4 mg/L, 8 mg/L, and 500 mg/L respectively (Fig. 3 and Table 1). This trend clearly indicates that the CMC is strongly dependent on the hydrophobic content in the chain and MW of polymer. The CMC values reduce as the hydrophobic chain length increased in the polymer. This observation was true up to a certain extent of hydrophobic chain length. There are other molecular mechanics that drive the assembly of molecules into micelles which are discussed in detail in the sections below.

In this study, the CMC values were determined using a UV probe instead of fluorescent probes such as pyrene, because of interference from resveratrol in fluorescent measurements. Resveratrol has very broad excitation (290–350 nm) and emission (380–420 nm) ranges. These broad ranges overlap with those of fluorescent probes such as pyrene. The dimethyl yellow employed in this study has poor aqueous solubility (13 mg/L), which provides an opportunity to study the UV absorption of dimethyl yellow under a particular wavelength and monitor the change in wavelength of the maximum absorption as a function of surfactant or amphiphilic polymer concentration [27]. The CMC values obtained by both dye solubilisation and wavelength shift have correlated well. This indicates efficiency of the dimethyl yellow as an alternative UV probe in CMC determination.

The micelles size and polydispersibility index are given Table 2. There was no exact correlation between micelle size and polymer architecture such as chain length and hydrophobic content. In general, the polymer conjugates with less hydrophobic content formed micelles with a smaller diameter. The micelles diameter increased with an increase in polymer concentration. This could be due to the formation of large aggregates at higher concentrations.

**Table 2:** Polymeric micelle size and polydispersibility index of resveratrol-polymer conjugates.

Concentration of polymer (%)	Size nm (SD)	Polydispersibility index (SD)
**Meo-PEG-PLAO-Succ RSV 2 kDa**		
0.05	170 (0.6)	0.21 (0.05)
0.1	249 (10.4)	0.59 (0.03)
0.2	255 (3.0)	0.49 (0.08)
**Meo-PEG-PLAO-Succ RSV 6.6K**		
0.05	147(3)	0.25(0.08)
0.1	147(6.1)	0.34(0.07)
0.2	165(12.4)	0.75 (0.15)
**MeO-PEGO-Succ-RSV 2 kDa**		
0.1	116 (1.5)	0.12 (0.02)
0.25	136 (1.2)	0.08 (0.05)
0.5	181 (2.5)	0.19 (0.03)

**Stability of polymer conjugates in buffer.** The stability of the polymer conjugates was studied in buffers of pH 4.5 and 7.4 at 37°C, physiologically relevant to the pH of lysosomes and blood. All the polymer conjugates of resveratrol were found to be stable in pH 4.5, with nearly 90% of the polymer conjugates remaining intact after 24 h (Table 1). The **MeO-PEGN-Succ-RSV** 2 kDa and 20 kDa, were found to be unstable at pH 7.4 with only about 9% polymer conjugates remaining intact after 24 h, which indicates that the ester link is unstable in slightly alkaline conditions. Similar results were reported for PEG-scutellarin conjugates and PEG-Taxol conjugates [23,39]. Contrary to this, **MeO-PEGO-Succ-RSV** 2 kDa was found to be relatively stable in pH 7.4 with nearly 65% of polymer conjugate found intact after 24 hours. This could be attributed to self assembly of polymer conjugates to micelles. The micelles would form a barrier against hydrolysis of esters. This was further confirmed from results of micelle forming **MeO-PEG-PLAO-Succ-RSV** conjugates which were found to be stable in pH 4.5 and 7.4 with > 78% polymer conjugates found intact after 24 h. As anticipated, the resveratrol-PEG ether **MeOH-PEG-RSV** 2 kDa did not hydrolyze in any of the buffers because of the stability of the ether linkage.

**Stability of resveratrol-polymer conjugates in rat plasma.** The effectiveness of the drug-polymer conjugates with an ester link for prolonged circulation in plasma would depend on the stability of the ester linkage in the plasma, as plasma esterase would facilitate hydrolysis. All PEG ester conjugates of resveratrol, **MeO-PEGN-Succ-RSV** 2 kDa and 20 kDa, and **MeO-PEGO-Succ-RSV** 2 kDa, irrespective of the linker and polymer chain length, hydrolysed rapidly to yield free resveratrol when incubated with rat plasma. It is interesting to note that whatever amount of resveratrol released after hydrolysis was *trans* isomer. There was no isomeric conversion. The hydrolysis was so rapid that almost no intact polymer conjugate was detected by HPLC at the initial time point of 0 h. The rate of hydrolysis was more rapid than that reported for campothecin-20-PEG and taxol-2’-PEG which have plasma t_½_ of 2 and 0.4 h respectively [23]. The reason for this rapid hydrolysis is presumably a result of the more reactive phenolic-ester linkage involved in the PEG-RSV conjugates. Although there are a few reports suggesting the application of more electronegative acid linkers, such as glycine for campothecine and peptides in the case of doxorubicin, results in greater plasma stability, for resveratrol, PEGylation via a glycine linker did not improve plasma stability in previous studies [40]. To improve the plasma stability of our conjugates, micelle-forming polymeric conjugates were explored. Accordingly the **MeO-PEG-PLAO-Succ-RSV** of different MW and with a PLA/PEG MW ratio of 1:0.75 and 4.6:2, were prepared and evaluated for plasma stability. Their plasma t_1/2_, MW of conjugate, MW of PLA/PEG segments are shown in Table 1. The results indicate that **MeO-PEG-PLAO-Succ-RSV** 2 kDa was more stable than the **MeO-PEG-PLAO-Succ-RSV** 6.6kDa conjugate. The other micelle forming conjugate **MeO-PEGO-Succ-RSV** 2 kDa was no different to **MeO-PEGN-Succ-RSV** 2 and 20 kDa in terms of plasma stability despite its better stability in the buffer. It hydrolyzed rapidly with no intact conjugate being detected at the initial time point.

The plasma stability of the polymeric drug ester conjugate is a complex phenomenon. The enzymatically catalyzed release of various drugs bound to N-(2-hydroxypropyl)methacrylamide (HPMA)copolymers via ester linkages was found to be dependent on many factors, including inter- and intra-molecular forces and the hydrophobicity of the drug and polymer [41–43]. Similarly, studies conducted to understand colloidal stability, protein adsorption and phagocytic clearance of PEG-PLA nanostructures, revealed that aggregation into colloidally stable micelles/nanoparticles was most favored when the PEG corona (PEG chain length and surface density) is high [44–47]. Higher PLA content in the PEG-PLA chain has been reported to be associated with decreased colloidal stability and reduces tendency to aggregate into micelles. In addition, higher protein adsorption and phagocytic clearance was observed due to "naked" PLA patches on the micelles surface [45]. Considering these observations and reports, it can be concluded that to improve plasma stability of the conjugate, there has to be an optimum hydrophobicity, micelle forming ability and colloidal stability, with adequate surface protection by the PEG. In this regard, presumably **MeO-PEG-PLAO-Succ-RSV** 2 kDa had relatively optimum hydrophobicity, forming compact and stable micelles with a relatively strong PEG corona, thereby providing maximum stability against ester hydrolysis. **MeO-PEG-PLAO-Succ-RSV** 6.6kDa, on other hand, with a longer PLA chain length, presumably lead to the formation of micelles with significantly higher "naked" PLA surfaces thus providing less protection against ester hydrolysis. Consistent with these assumptions, AFM/FESEM of air dried samples of **MeO-PEG-PLAO-Succ-RSV** 2 kDa micelles revealed formation of solid nanoparticle-like structures and these solid nanostructures could impart additional resistance to enzymatic hydrolysis. The resveratrol-PEG ethers were found to be intact after 5 h incubation with rat plasma, as anticipated because the ether linkage is resistance to enzymatic cleavage.

**Fig 4:**
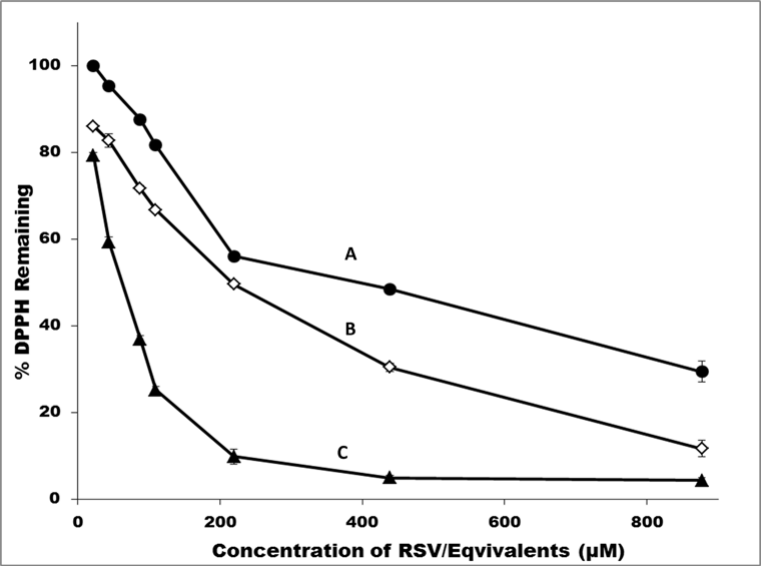
DPPH inhibition profile of A: Resveratrol; B: MeO-PEGN-Succ-RSV, 2kDa; C: MeO-PEG-RSV ether, 2kDa All the data are presented as mean ± SD (n = 3). Mw quoted is for polymer only. Mw for RSV is 228 Da.

***In vitro* antioxidant assay.** The *in vitro* antioxidant profile as assessed by DPPH inhibition assay is shown in Fig. 4. The IC 50 value of resveratrol was found to be 65 μM, which is in close agreement with the value reported by Fauconneau *et al*. [48]. The IC 50 value for resveratrol-PEG ether **MeO-PEG-RSV**, 2 kDa was 2190 μM (equal to 219 μM of resveratrol) and the values were similar for **MeO-PEGN-Succ-RSV** 2 kDa and **MeO-PEG-PLAO-Succ-RSV** 2 kDa. The profile shows that resveratrol conjugated to polymers is less active than native resveratrol with regard to antioxidant activity (Fig. 4). There was an almost 3.5 times increase in the IC 50 values when compared to resveratrol. It is, however, anticipated that the resveratrol polymer ester conjugates would hydrolyze in the body slowly releasing native resveratrol. The decrease in antioxidant activity indicates that all the hydroxyl groups of resveratrol are involved in reaction with free radicals. Hence, blocking one or more hydroxyl groups would lead to a decrease in activity. The antioxidant activity measured by this assay method was based on direct neutralization of free radicals and may not reflect actual *in vivo* activity, as resveratrol exerts *in vivo* antioxidant activity by many other mechanisms. For instance, it was reported that resveratrol could mediate the release or production of nitric oxide, which has stronger affinity for O_2_^-^[1]. Resveratrol is believed to induce enzymes dismutase and NAD(P)H:quinineoxidoreductase, both of which scavenge the reactive oxygen species [1].

To produce a successful clinical candidate, there has to be a balance of all biological properties such as activity, toxicity and metabolism. The best molecule, in terms of biological activity would not necessarily make it to clinical studies or to the market due to issues such as metabolism [49]. Similarly, having an unmodified resveratrol with higher activity but prone to metabolism would not be useful for clinical studies. Chemically conjugated resveratrol, such as the resveratrol-PEG ether **MeO-PEG-RSV**, with a hydrolytically stable link and prolonged plasma residence time and better pharmacokinetic profile would offer an alternative choice to resveratrol despite the moderate decrease in the antioxidant activity.

**Fig 5:**
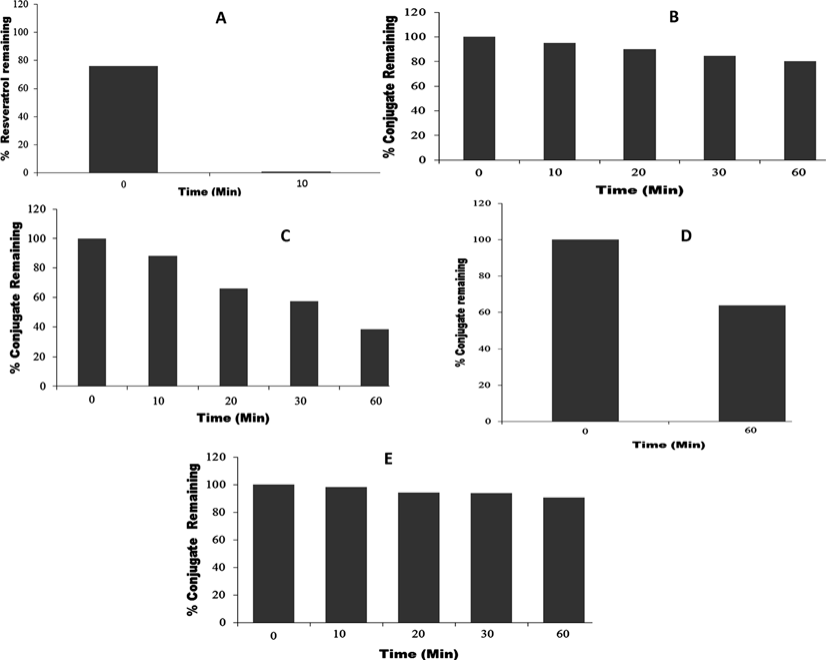
The degradation profile of A: Resveratrol and B: MeO-PEG-PLAO-Succ-RSV 2 kDa in microsomal incubations with UDPA. C: MeO-PEGN-Succ-RSV 2 kDa in microsomal incubation, D: MeO-PEGN-Succ-RSV 2 kDa in incubation buffer without microsomes. E: MeO-PEG-RSV ether 2 kDa in microsomal incubation. Mw quoted is for polymer only. Mw for RSV is 228 Da.

**Fig 6:**
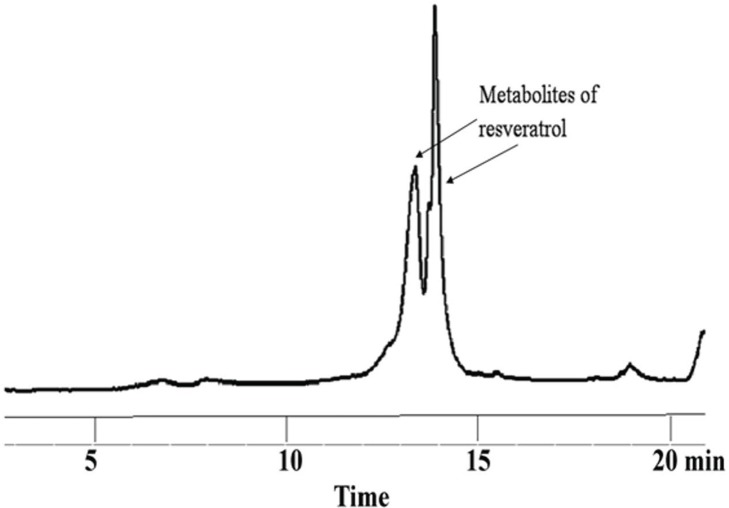
Chromatogram of resveratrol in microsomal incubations at 10 minutes.

***In vitro* metabolism studies of resveratrol and its polymer conjugates.** The metabolism studies conducted by incubation with rat liver microsomes in the presence of the UGT reaction mixture showed that resveratrol readily underwent metabolism to produce two metabolites, presumably glucuronides, and the retention times of those metabolites correspond to those observed in rat plasma samples, degradation pattern is shown Fig. 5 and metabolites in Fig. 6. The metabolism of resveratrol was rapid and almost all of the resveratrol was metabolized within 10 minutes. The incubations of **MeO-PEG-PLAO-Succ-RSV** 2 kDa with microsomes over 60 minutes did not result in any detectable level of metabolites by HPLC and presumably there was no metabolism occurring within the test period. The incubation samples of **MeO-PEGN-Succ-RSV** 2 kDa showed formation of moderate amount of glucuronide metabolites. However the control incubations of **MeO-PEGN-Succ-RSV** 2 kDa without microsomes showed a significant degree of conjugate hydrolysis to free resveratrol which could be due to pH. The resveratarol generated in such a way would have been subsequently metabolized in the incubations containing microsomes under similar pH conditions. In contrast, the resveratrol-PEG ether **MeO-PEG-RSV** 2 kDa was stable in microsomal incubations (Fig. 5E). These observations clearly demonstrate that to provide protection against the metabolism of resveratrol, the polymer conjugates need to be intact and hydrolyze slowly in the body.

**Fig 7:**
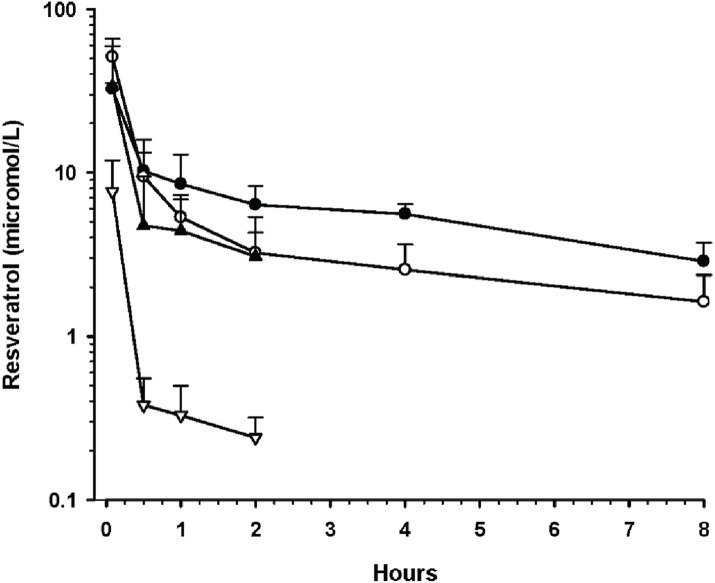
Plasma concentration-time profiles for resveratrol formulations in rats. Doses are resveratrol equivalents and data are mean ± SD. Symbols:MeO-PEG-RSV ether 2 kDa, 2 mg/kg (●; n = 5); MeO-PEG-RSV ether 5 kDa, 2 mg/kg (◯; n = 6); MeO-PEG-PLAO-Succ-RSV 2kDa, 2 mg/kg (▲; n = 6); Resveratrol, 10 mg/kg (▽; n = 4).

Single dose intravenous pharmacokinetic studies. The plasma concentration profiles of resveratrol, **MeO-PEG-PLAO-Succ-RSV** 2 kDa, RSV-PEG ethers **MeO-PEG-RSV** 2 kDa and **MeO-PEG-RSV**5 kDa are presented in Fig. 7. After intravenous dosing of resveratrol at 10 mg/kg, the plasma concentration profile showed rapid elimination of resveratrol from the plasma. There were four major metabolites detected by HPLC and characterized by LC-MS/MS. The four metabolites were identified and assigned to 3- and 4’- monoglucuronides, 3- and 4’- mono sulphates [28].

Pharmacokinetic parameters for the resveratrol formulations are shown in Table 3, The elimination half-life (t_½_ = 1.4 h) and clearance (CL = 20 L/h/kg) were comparable to previous reports (t_½_ = 1.3 h and CL = 11–19 L/h/kg [5, 9, 50]. After two hours, the resveratrol concentration was below the limit of detection, indicating its rapid metabolism. By comparison, **MeO-PEG-PLAO-Succ-RSV** 2 kDa was given to rats at one-fifth of the resveratrol dose (2 mg/kg equivalent), but resulted in a 9-fold higher AUC, a 34-fold lower apparent CL and 24-fold lower V_ss_, suggesting a reduced tissue distribution of the drug that should lead to more persistent retention of the drug in the circulation. However, the lack of detectable conjugate after 2 hr can be attributed to clearance by the mononuclear phagocyte systems (MPS) [47].

**Table 3:** Pharmacokinetic parameters for resveratrol and its conjugate formulations in rats.

	AUC_0-∞_		t_½_		MRT^‡^		CL^‡^		V_ss_ ^‡^	
	(μmol.h/L)	P value, 95% CI	(h)	P value, 95% CI	(h)	P value, 95% CI	(L/h/kg)	P value, 95% CI (Significance)	(L/kg)	P value, 95% CI (Significance)
**MeO-PEG-RSV ether 5 kDa; 2 mg/kg^†^ (n = 6)**	50 ± 15	0.0003***	5.3 ± 2.1	0.0086**	5.9 ± 3.5	0.0361*	0.19 ± 0.06	0.0003***	1.01 ± 0.41	0.009**
**MeO-PEG-RSV ether 2 kDa; 2 mg/kg^†^ (n = 5)**	74 ± 16	0.0001****	5.5 ± 1.4	0.015**	7.1 ± 2.0	0.0012**	0.12 ± 0.02	0.001***	0.87 ± 0.28	0.0170*
**MeO-PEG-PLAO-Succ-RSV 2kDa; 2 mg/kg^†^ (n = 6)**	21 ± 17	0.0645	1.7 ± 0.5	0.512	1.9 ± 0.4	0.214	0.58 ± 0.26	0.0004***	1.12 ± 0.60	0.0094**
**Resveratrol; 10 mg/kg^†^ (n = 4)**	2.4 ± 0.7	Not applicable	1.4 ± 0.9	Not applicable	1.3 ± 1.0	Not applicable	20.1 ± 8.4	Not applicable	26.8 ± 19.1	Not applicable

^†^ 2 mg/kg = 8.8 μmol/kg; 10 mg/kg = 44 μmol/kg. Doses are resveratrol equivalents^†^ and data are mean ± SD.

^‡^ MRT: mean residence time;

CL: clearance; V_ss_: volume of distribution at steady-state.

P values are from comparison of a particular group against resvertrol,

* ** *** **** indicate p <0.05, increasing number of stars implies increasing significance.

The plasma profile of **MeO-PEG-PLAO-Succ-RSV** 6.6 kDa was considerably different to that of **MeO-PEG-PLAO-Succ-RSV** 2 kDa (data not shown). There was no quantifiable polymer conjugate detected in the plasma, even at initial time point of 5 min. The rapid decline or non-availability of polymer conjugate could be due to the combined effect of hydrolysis and MPS clearance. Unlike **MeO-PEG-PLAO-Succ-RSV** 2 kDa, **MeO-PEG-PLAO-Succ-RSV** 6.6 kDa displayed a higher level of metabolites, possibly due to more "naked" PLA on the surface of micelles, which in turn made the conjugate more vulnerable to enzymes and MPS attack. This lead to hydrolysis of ester and released resveratrol. The latter was subsequently metabolized resulting in a high level of metabolites. These observations correlate with results obtained from the *in vitro* plasma stability study.

The resveratrol-PEG ethers **MeO-PEG-RSV**2 kDa and **MeO-PEG-RSV** 5 kDa had a higher AUC and lower apparent CL compared to **MeO-PEG-PLAO-Succ-RSV** 2kDa, but the V_ss_ was comparable (Table 3). When compared to the resveratrol group, these two conjugates are statistically significantly (P<0.05) increased in mean residence time, AUC, volume of distribution, clearance and elimination half-life. The prolonged residence time of resveratrol-PEG ether conjugates in the blood circulation could be attributed to the combined effect of PEG conjugation and ether linkage, which increased the hydrophilicity of resveratrol and avoided the phagocyte detection and metabolism. However, eventually the conjugates underwent filtration via the glomerulus. The similarity in pharmacokinetic parameters for resveratrol-PEG ether conjugates (**MeO-PEG-RSV** 2 kDa and **MeO-PEG-RSV** 5 kDa) suggests that an increase in MW from 2 kDa to 5 kDa has a marginal effect on kidney filtration [17]. In a previous report, pharmacokinetic studies with radio labelled PEGs of various MW clearly showed that a significant effect of MW on clearance of PEG can be observed only above a MW of 20 kDa [51]. Nevertheless, it is plausible that the special conformational arrangements of 3,4’-di(PEG) may give a more hydrodynamic volume and low kidney filtration ^13^. Indeed, Fee *et al*. have studied the size comparison between proteins PEGylated with linear and branched chain PEGs [52] and concluded that branched PEG would have a higher viscosity and larger radius than proteins PEGylated with linear PEGs of the same molecular weight. Based on the same reasoning, di-substituted resveratrol with a PEG of 2 kDa could have a branched structure and a more hydrodynamic volume than mono-substituted resveratrol with PEG of 5 kDa. However these findings need further confirmation using suitable analytical techniques. Overall, the pharmacokinetic profile of resveratrol-PEG ethers is encouraging and further improvement in the pharmacokinetic properties may be achieved by increasing the MW of PEG.

## Conclusion

Ester and ether-based PEG and PEG-PLA polymer conjugates (soluble and polymeric micelles) to improve the pharmacokinetic profile of resveratrol were synthesized and evaluated. Studies of the buffer stability, plasma stability and *in vitro* metabolism using rat liver microsomes, and pharmacokinetics in rat showed that **MeO-PEG-PLA-Succ-RSV** 2 kDa, RSV-PEG ethers 2 kDa and 5 kDa consistently out-performed other polymers such as PEG linked to resveratrol via ester bond and resveratrol itself. It was shown that both the type of linkage between the polymer and drug, and the relatively compact PEG corona on the polymeric micelles are crucial for rendering a stable conjugate system which protects from degradation. For the polymers containing an ester bond, it is the ability to form micelles that enhances *in vitro* and *in vivo* stability. In conclusion, our study demonstrates that polymeric conjugates can be an effective approach to improve the pharmacokinetic profile of resveratrol. Furthermore, the polymer architecture and linkage can have a strong influence on the stability of conjugates *in vitro* and *in vivo*.

NMR spectra and structures of polymer conjugates are given in S1 File. This material is available free of charge via the Internet at http://www.plosone.org

## Supporting Information

S1 File Fig. A, ^1^H NMR spectra (400 MHz) for: (a) MeO-PEGN-SuccOH; (b) the resveratrol-PEG conjugate mixture from the synthesis of MeO-PEGN-Succ-RSV; (c) resveratrol; and (d) the solution from (b) after treatment with water and heat (90°C) for *ca*. 1 week). All solutions are in *d*_6_-DMSO. Key: † = *d*_5_-DMSO, ‡ = H_2_O. To identify the low intensity down-field signals the spectra in (a), (b) and (d) have been expanded vertically. **Fig. B,** The down field region of the ^1^H NMR spectra (400 MHz) for: (a) a solution of the resveratrol-PEG conjugate mixture in *d*_6_-DMSO showing the assigned signals for the two major products **4'-MeO-PEGN-Succ-RSV** and **3-MeO-PEGN-Succ—RSV**; and (b) a solution of resveratrol in *d*_6_-DMSO. **Fig. C,** ^1^H NMR spectra for the MeO-PEGN-Succ-RSV conjugate mixture in *d*_6_-DMSO, recorded at 400 MHz. Key: † = *d*_5_-DMSO, ‡ = H_2_O. **Fig. D,** ^1^H NMR spectra for MeO-PEGN-Succ-OH in *d*_6_-DMSO, recorded at 400 MHz. Key: † = *d*_5_-DMSO, ‡ = H_2_O. **Fig. E,** ^1^H NMR spectra of the MeO-PEGN-Succ-RSV conjugate mixture after treatment with water and heat (90°C) for *ca*. 1 week, in *d*_6_-DMSO, recorded at 400 MHz. Key: † = *d*_5_-DMSO, ‡ = H_2_O.
(DOCX)
