# Supplementary material for: Stabilization of Resveratrol in Blood Circulation by Conjugation to mPEG and mPEG-PLA Polymers: Investigation of Conjugate Linker and Polymer Composition on Stability, Metabolism, Antioxidant Activity and Pharmacokinetic Profile
Source: PLoS One. 2015 Mar 23;10(3):e0118824. doi: 10.1371/journal.pone.0118824 (PMC4370505; doi:10.1371/journal.pone.0118824)
Supplement: S1 File — All solutions are in d 6-DMSO. Key: † = d 5-DMSO, ‡ = H2O. To identify the low intensity down-field signals the spectra in (a), (b) and (d) have been expanded vertically. Fig. B, The down field region of the 1H NMR spectra (400 MHz) for: (a) a solution of the resveratrol-PEG conjugate mixture in d 6-DMSO showing the assigned signals for the two major products 4'-MeO-PEGN-Succ-RSV and 3-MeO-PEGN-Succ—RSV; and (b) a solution of resveratrol in d 6-DMSO. Fig. C, 1H NMR spectra for the MeO-PEGN-Succ-RSV conjugate mixture in d 6-DMSO, recorded at 400 MHz. Key: † = d 5-DMSO, ‡ = H2O. Fig. D, 1H NMR spectra for MeO-PEGN-Succ-OH in d 6-DMSO, recorded at 400 MHz. Key: † = d 5-DMSO, ‡ = H2O. Fig. E, 1H NMR spectra of the MeO-PEGN-Succ-RSV conjugate mixture after treatment with water and heat (90°C) for ca. 1 week, in d 6-DMSO, recorded at 400 MHz. Key: † = d 5-DMSO, ‡ = H2O. (DOCX) [file pone.0118824.s001.docx]

**Stabilization of resveratrol in blood circulation by conjugation to mPEG and mPEG-PLA polymers: investigation of conjugate linker and polymer composition on stability, metabolism, antioxidant activity and pharmacokinetic profile.**

*Basavaraj Siddalingappa^1,3*^, Heather A. E. Benson^*^, David H. Brown^2^, Kevin T. Batty^1^, Yan Chen^1^*

**^1^**School of Pharmacy, CHIRI-Bioscience, Curtin University, GPO Box U1987 Perth, Western Australia

^2^Department of Chemistry, Curtin University, GPO Box U1987 Perth, Western Australia.

^3^Present Address, Graduate College of Biomedical Sciences, Western University of Health Sciences, Pomona, CA, USA.

Corresponding author: Dr.Basavaraj Siddalingappa, Graduate College of Biomedical Sciences, Western University of Health Sciences, Pomona, CA 91766, USA. Email: [bsiddalingappa@westernu.edu](mailto:bsiddalingappa@westernu.edu); Tel: +1 909 469 6476

.


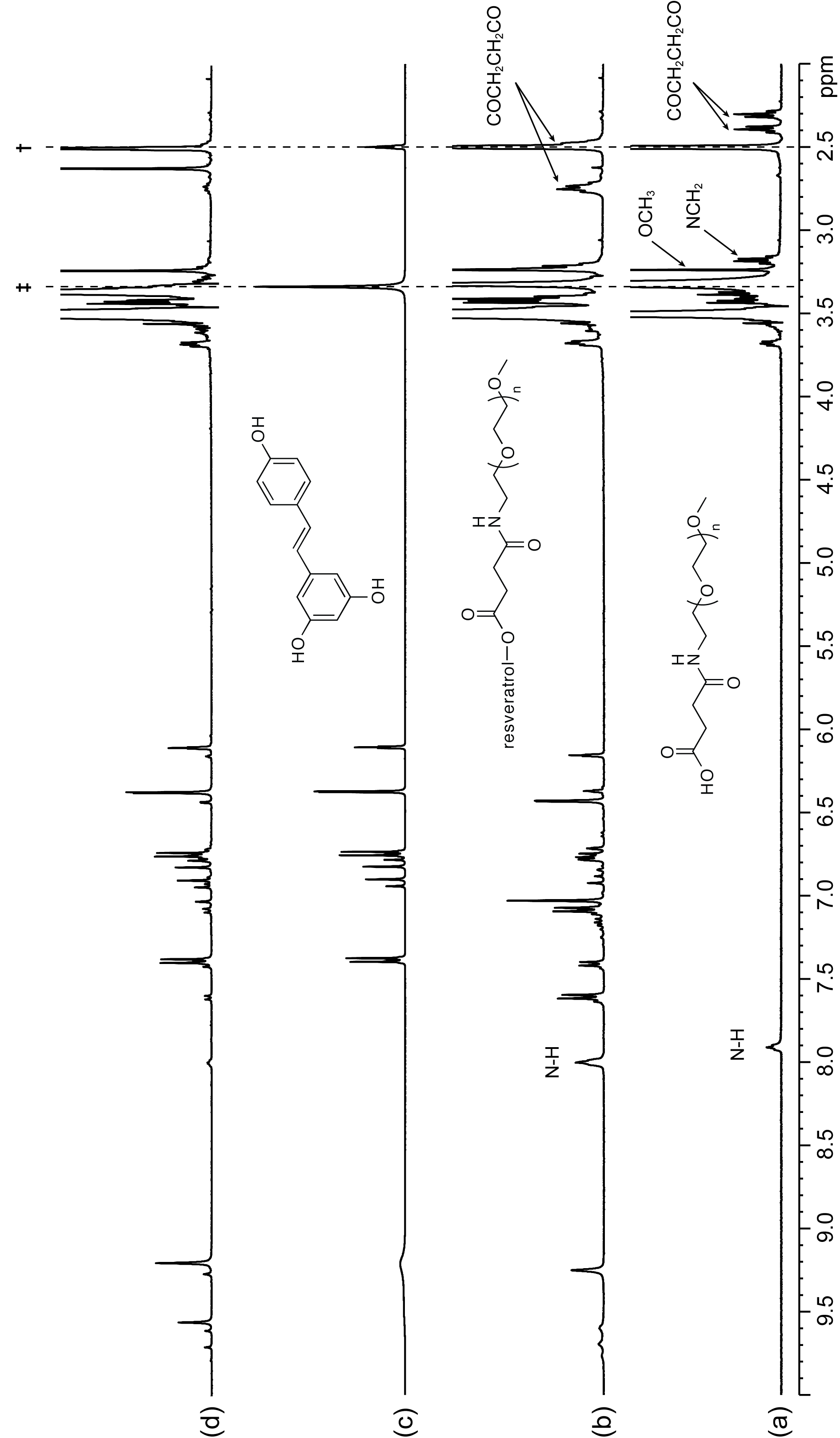


**Figure A**. ^1^H NMR spectra (400 MHz) for: (a) MeO-PEGN-SuccOH; (b) the resveratrol-PEG conjugate mixture from the synthesis of MeO-PEGN-Succ-RSV; (c) resveratrol; and (d) the solution from (b) after treatment with water and heat (90 °C) for *ca.* 1 week). All solutions are in *d*_6_-DMSO. Key: † = *d*_5_-DMSO, ‡ = H_2_O. To identify the low intensity down-field signals the spectra in (a), (b) and (d) have been expanded vertically.


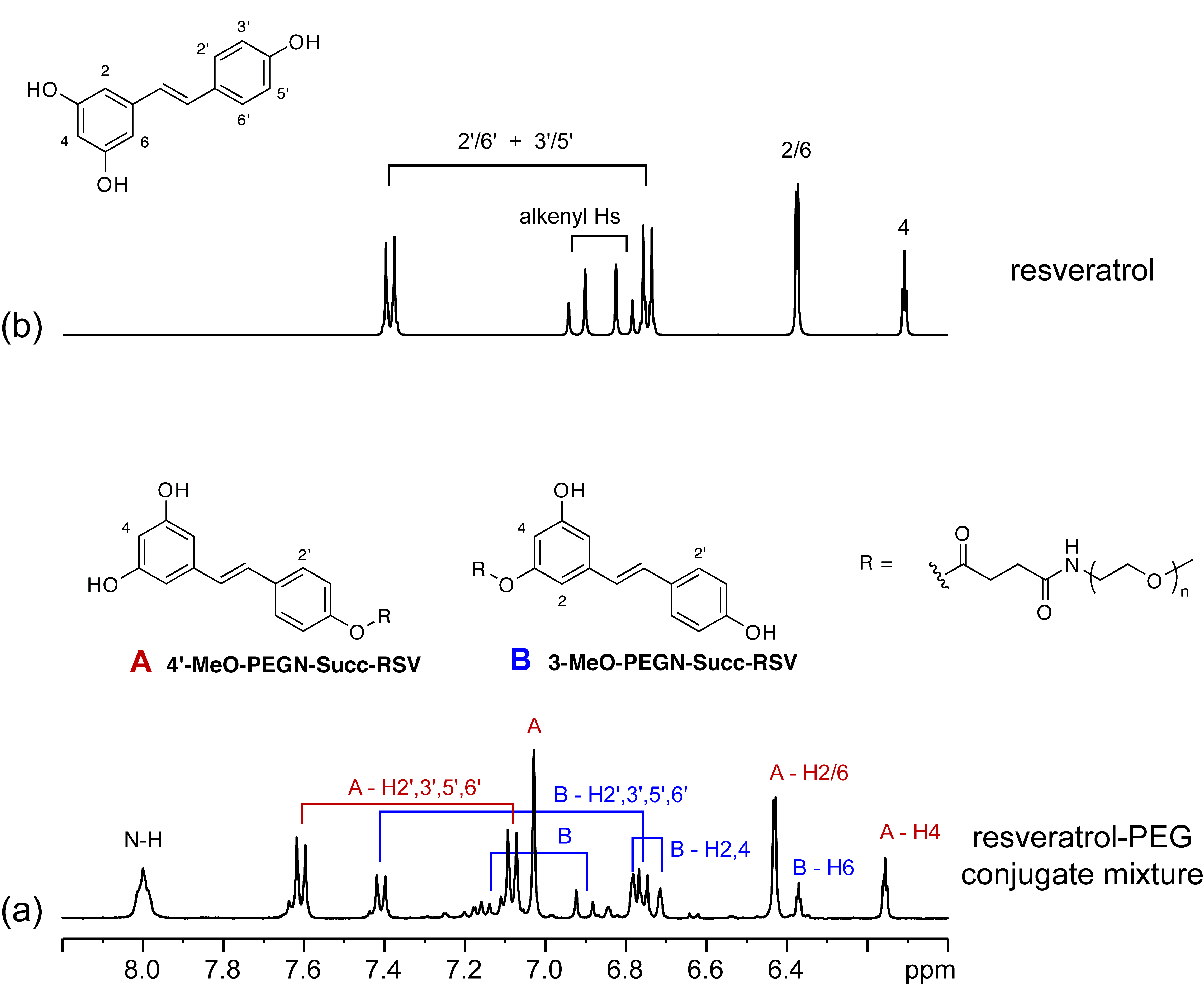


**Figure B**. The down field region of the ^1^H NMR spectra (400 MHz) for: (a) a solution of the resveratrol-PEG conjugate mixture in *d*_6_-DMSO showing the assigned signals for the two major products **4'-MeO-PEGN-Succ-RSV** and **3-MeO-PEGN-Succ--RSV**; and (b) a solution of resveratrol in *d*_6_-DMSO.


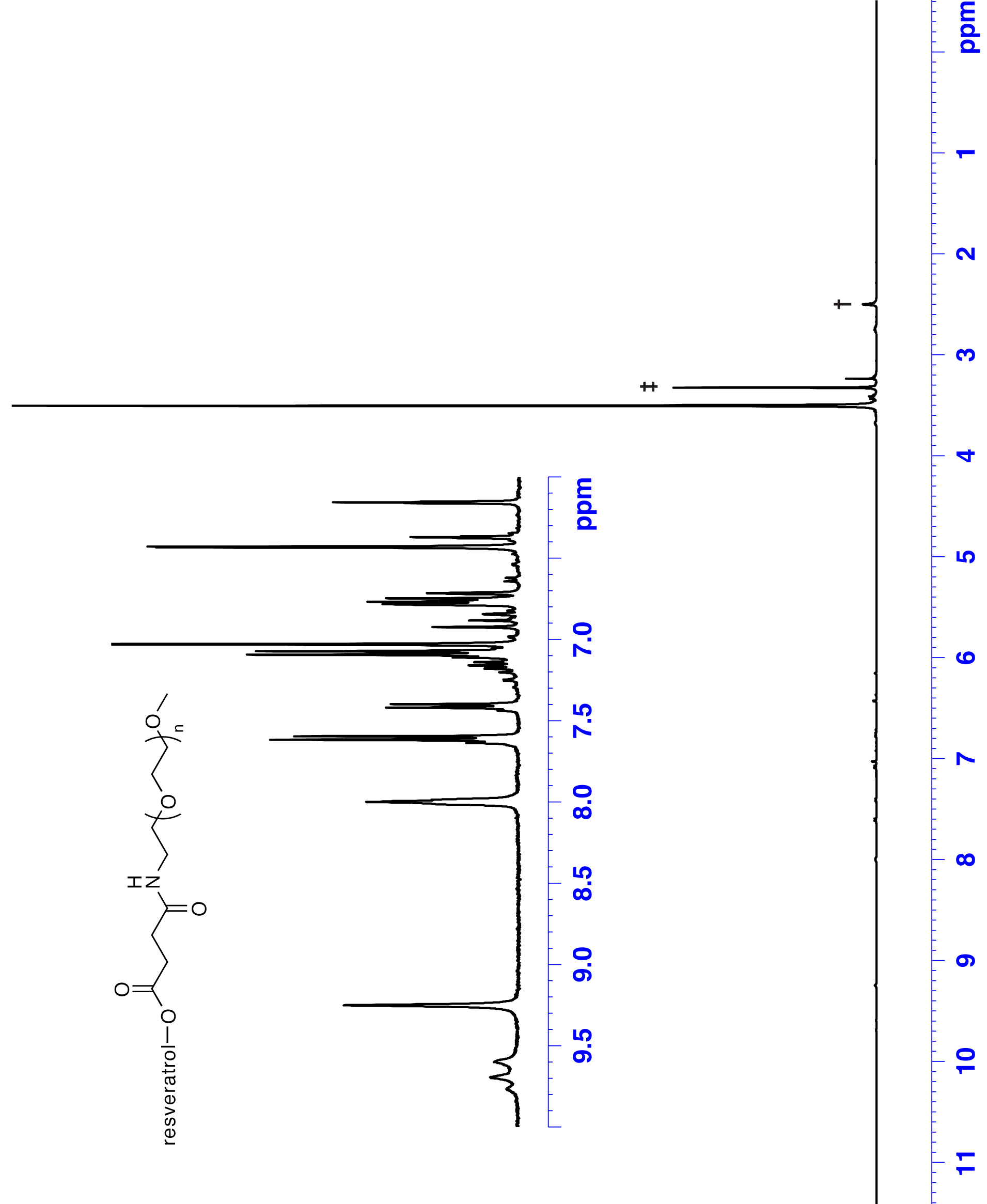


**Figure C**. ^1^H NMR spectra for the MeO-PEGN-Succ-RSV conjugate mixture in *d*_6_-DMSO, recorded at 400 MHz. Key: † = *d*_5_-DMSO, ‡ = H_2_O.


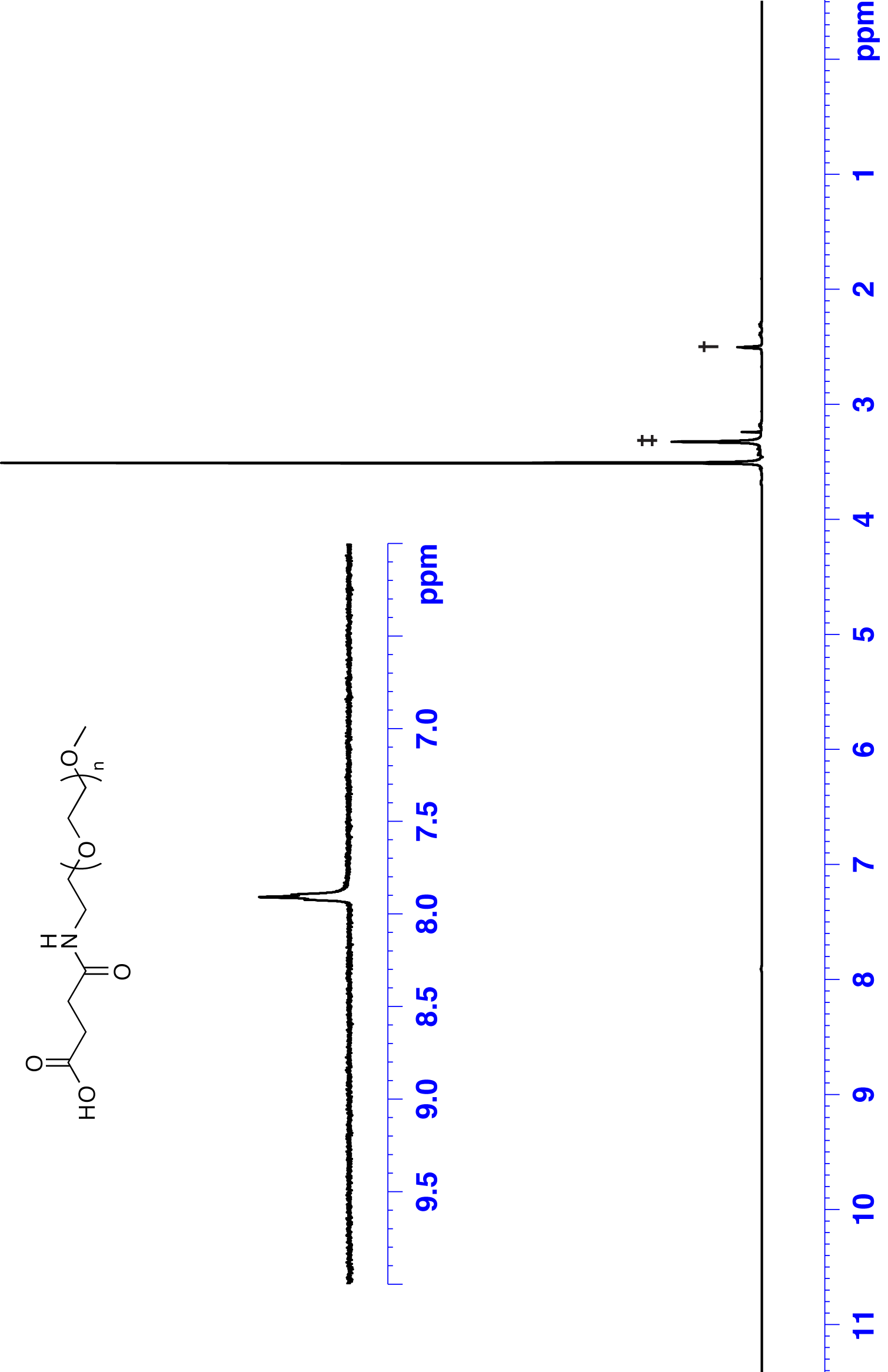


**Figure D**. ^1^H NMR spectra for MeO-PEGN-Succ-OH in *d*_6_-DMSO, recorded at 400 MHz. Key: † = *d*_5_-DMSO, ‡ = H_2_O.


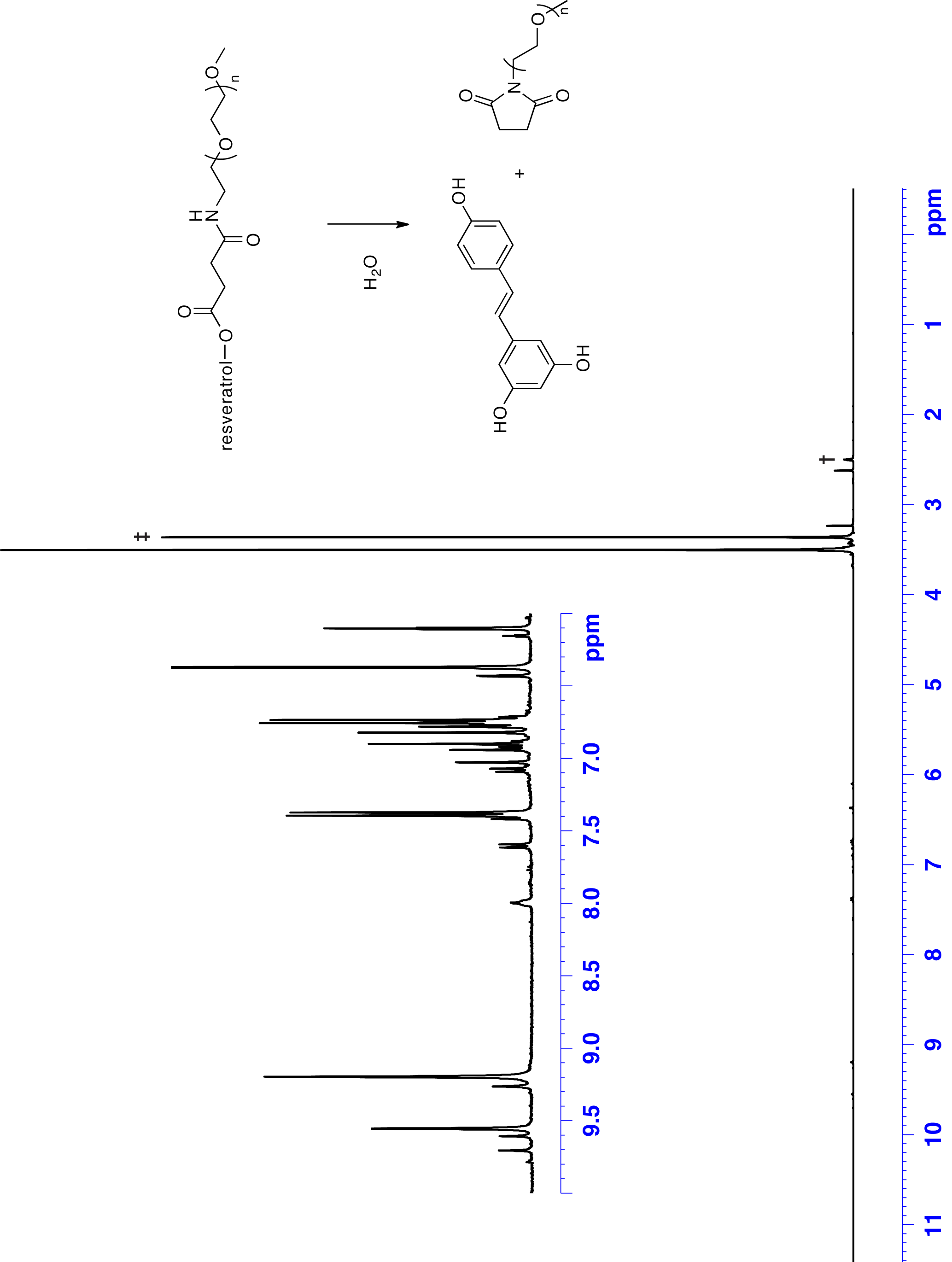


**Figure E**. ^1^H NMR spectra of the MeO-PEGN-Succ-RSV conjugate mixture after treatment with water and heat (90 °C) for *ca.* 1 week, in *d*_6_-DMSO, recorded at 400 MHz. Key: † = *d*_5_-DMSO, ‡ = H_2_O.
